# Lactate levels after major cardiac surgery are associated with hospital length of stay

**DOI:** 10.1186/cc14273

**Published:** 2015-03-16

**Authors:** LW Andersen, M Holmberg, P Patel, KM Berg, M Cocchi, M Doherty, K Khabbaz, MW Donnino

**Affiliations:** 1 Beth Israel Deaconess Medical Center, Boston, MA, USA

## Introduction

The objective of the study was to evaluate whether postoperative lactate values are associated with hospital length of stay in patients undergoing major cardiac surgery. Previous studies have shown an association between postoperative lactate levels and increased morbidity and mortality after major cardiac surgery. However, the association between lactate and hospital length of stay has not been adequately characterized.

## Methods

We performed a retrospective analysis of all patients presenting for coronary artery bypass grafting and/or valve surgery between 2002 and 2014 at a tertiary care center in Boston, who had a lactate level measured within 3 hours of skin closure. Lactate values were categorized into clinical meaningful categories: 0 to 2 mmol/l (normal), 2 to 4 mmol/l (elevated) and ≥4 mmol/l (high) to allow for nonlinear effects. The unadjusted association between lactate group and length of stay was assessed with the Kruskal-Wallis test and *post hoc *Wilcoxon rank-sum tests. To assess the association between postoperative lactate levels and hospital length of stay we performed multivariable Poisson regression with robust variance estimates. We adjusted for more than 30 variables including patient demographics, comorbidities, cardiac characteristics (for example, New York Heart Association class and ejection fraction), and surgical characteristics (for example, year, status (elective, urgent, emergent), type of procedure, perfusion time, and cross clamp time).

## Results

We included a total of 1,267 patients. The median age was 68 (quartiles: 59, 76), 32% were female, 68% underwent coronary artery bypass grafting and 59% underwent valve surgery. Median length of hospital stay was 6 days (quartiles: 5, 9). Median length of stay in the normal, elevated and high lactate groups were 5 days (quartiles: 4, 7), 6 days (quartiles: 5, 9) and 9 days (quartiles: 6, 17), *P *< 0.001 for comparison. In multivariable analysis, patients with an elevated lactate had a 1.12 times (95% CI: 1.02 to 1.23, *P *= 0.02) longer length of stay compared with those with normal lactate. Patients with a high lactate had a 1.30 times (95% CI: 1.10 to 1.53, *P *= 0.002) longer length of stay compared with those with normal lactate.

## Conclusion

Postoperative lactate levels are associated with increased length of hospital stay in patients undergoing major cardiac surgery. Interventions aimed at decreasing postoperative lactate levels may decrease hospital length of stay.

